# Longitudinal Management of Post-traumatic Brain Injury Bipolar-Spectrum Disorder With Low-Dose Oral Glutamatergic Augmentation: Extended Follow-Up Revealing Induction, Overshoot, Stabilization, and Transition to PRN Maintenance

**DOI:** 10.7759/cureus.108595

**Published:** 2026-05-10

**Authors:** Ngo Cheung

**Affiliations:** 1 Psychiatry, Cheung Ngo Medical Limited, Hong Kong, HKG

**Keywords:** bipolar, plasticity, psychiatric pharmacology, synaptic pruning, traumatic brain injury(tbi)

## Abstract

Bipolar-spectrum illness emerging after traumatic brain injury (TBI) can be difficult to treat and may present with mixed or agitated depressive features that appear sensitive to glutamatergic modulation. In post-TBI cases, diagnostic certainty is often limited because irritability, impulsivity, sleep disturbance, affective lability, and cognitive change may overlap with frontal-limbic injury syndromes. This case is, therefore, framed as probable bipolar-spectrum disorder secondary to TBI rather than definitive idiopathic bipolar disorder. The Cheung Glutamatergic Regimen (CGR)--low-dose dextromethorphan with CYP2D6 inhibition plus piracetam--is used here only as a shorthand for an open-source, free-to-use, non-proprietary combination of off-patent components, not as a branded product. This report describes a woman in her mid-thirties with right frontal atrophy after a 2009 subdural hematoma who later developed probable bipolar-spectrum illness. On 21 October 2025, she presented with severe depressive relapse, insomnia, persistent rumination, irritability, and hypnagogic phenomena, with a Patient Health Questionnaire-9 (PHQ-9) score of 22. After partial improvement on valproate, risperidone, and Deanxit, dextromethorphan 30 mg nightly and piracetam 600 mg nightly were added on 5 November 2025. Within weeks, rumination decreased and mental flexibility improved, but transient mild hypomanic or frontal-disinhibition-like symptoms emerged, especially inappropriate laughter with a moria-like quality. She self-reduced dextromethorphan to 22.5 mg, piracetam was increased, and euthymia returned. Over the next six months, PHQ-9 scores improved to 10-12 and Generalized Anxiety Disorder-7 (GAD-7) scores to 8-13, with functional gains including exercise and motorcycle riding lessons. Later medications included aripiprazole, paroxetine-controlled release, pregabalin, and low-dose quetiapine. By April 2026, dextromethorphan and piracetam were used as needed during stress-related or premenstrual dips. No further psychotic symptoms were reported, and later mild dissociative or cognitive complaints became manageable after dose adjustment. This single-patient course suggests a three-phase pattern: induction with a narrow therapeutic window and brief activation/overshoot, stabilization after titration, and later PRN maintenance. Dextromethorphan appeared temporally most linked to both clinical benefit and transient activation, while piracetam may have acted as a modulator. However, causal inference is limited by the uncontrolled design, early PHQ-9 improvement before CGR initiation, later polypharmacy, unmeasured pharmacokinetics, absence of standardized mania/cognitive measures, and incomplete PRN-frequency documentation. The case is also only hypothesis-generating in relation to transcriptomic findings implicating bipolar-specific plasticity-related biology. Low-dose oral glutamatergic augmentation may warrant study as a closely monitored induction and consolidation strategy in post-TBI bipolar-spectrum illness, but prospective controlled trials are needed before broader recommendations can be made.

## Introduction

Traumatic brain injury (TBI) is one of the strongest environmental risk factors for later bipolar disorder. Large registry studies from Denmark and Taiwan have reported a three- to five-fold increase in new-onset bipolar illness after moderate or severe head trauma [1,2]. When bipolar-spectrum symptoms follow TBI, the clinical picture often differs from idiopathic bipolar disorder. Patients may show more irritability, emotional lability, impulsivity, executive dysfunction, and migraine comorbidity, patterns thought to reflect chronic neuroinflammation and disruption of prefrontal-limbic circuits [3,4]. For that reason, post-TBI bipolar-spectrum disorder should be interpreted cautiously: some features may represent secondary mood dysregulation from structural frontal injury rather than idiopathic bipolar disorder. In this report, the formulation is, therefore, “probable bipolar-spectrum disorder secondary to TBI.”

Intravenous ketamine provided the clearest early proof that brief N-methyl-D-aspartate (NMDA) receptor blockade, followed by increased alpha-amino-3-hydroxy-5-methyl-4-isoxazolepropionic acid (AMPA) throughput and mechanistic target of rapamycin-dependent synaptogenesis, can relieve treatment-resistant depression and bipolar depression within hours [5-7]. In simpler terms, NMDA blockade may release downstream AMPA-mediated plasticity, while mTOR is one intracellular pathway linked to synapse formation. Even so, ketamine remains difficult to access for many patients because it requires supervised administration, can cause dissociative effects, and is costly; expert consensus also emphasizes the need for careful patient selection and monitoring [8]. That limitation has encouraged interest in oral approaches that may reproduce parts of the same mechanism.

One such approach combines dextromethorphan, a readily available NMDA antagonist with sigma-1 receptor agonist properties, with a cytochrome P450 2D6 inhibitor to slow metabolism and extend exposure. Peer-reviewed evidence for oral dextromethorphan-based glutamatergic approaches includes dextromethorphan-bupropion in major depressive disorder and dextromethorphan-quinidine in Alzheimer’s disease agitation, although these are not the same as the present regimen and cannot be used to infer efficacy in post-TBI bipolar-spectrum illness [9,10]. Piracetam is then added as a putative AMPA positive allosteric modulator to support the downstream plasticity cascade and possibly buffer excessive glutamatergic surges. Earlier peer-reviewed and preliminary reports have described clinically meaningful improvement with this combination or closely related oral glutamatergic strategies in refractory psychiatric presentations [11-13]. The full Cheung Glutamatergic Regimen (CGR), as previously described, may include optional L-glutamine; in the present case, the CGR exposure consisted of dextromethorphan and piracetam in the context of CYP2D6 inhibition, and L-glutamine was not used.

The abbreviation CGR is retained in this manuscript for clarity and continuity, but it is not a proprietary or patented label. It denotes a fully open-source, free-to-use clinical concept using existing off-patent agents, and the author makes no patent or licensing claim for the regimen. The term has also appeared in peer-reviewed publications, including The Journal of Clinical Psychiatry and Journal of Alzheimer’s Disease Reports [11,12].

The primary objective of this case report is to document the six-month clinical evolution, dose adjustments, adverse effects, functional changes, and transition from scheduled to pro re nata (PRN) use of low-dose oral dextromethorphan/piracetam augmentation in probable post-TBI bipolar-spectrum illness. The present report addresses the gap in longitudinal dosing information by describing a six-month observational course in a patient with well-documented post-TBI structural change and a clinically probable bipolar-spectrum presentation. The case shows distinct phases of response: an early induction period with transient hypomanic or disinhibition-like overshoot, later stabilization after dose adjustment, and eventual PRN use during predictable mood dips. It also shows how baseline features consistent with a mixed or agitated depressive state may shape both benefit and the width of the therapeutic window. These clinical observations could be considered alongside emerging cross-disorder transcriptomic findings that separate the molecular profile of bipolar disorder from that of major depressive disorder [14].

The working hypothesis was that, in patients with post-TBI bipolar-spectrum presentations and probable mixed features, low-dose oral glutamatergic augmentation could be associated with meaningful clinical improvement. At the same time, it was expected that treatment would require careful titration and might work best as an induction and consolidation tool rather than as indefinite daily therapy. Because this is a single uncontrolled case, the report is intended to generate hypotheses rather than establish efficacy, causality, or a generalizable treatment model.

## Case presentation

A mid-thirties woman whose psychiatric history was closely linked to a traumatic brain injury sustained in 2009. During an episode of domestic violence that year, she developed a right frontal subdural hematoma. Imaging at the time showed right frontal lobe atrophy, and a repeat MRI in 2019 confirmed that the atrophy had progressed. The original imaging studies were available only as hard copy at the time of the current presentation and were not scanned or digitally recorded; therefore, they could not be included in this report. No formal radiology report summary was available for extraction into the manuscript, which limits the reader’s ability to independently contextualize the neuroanatomical claim. According to family members, her personality and emotional regulation changed gradually over time in ways that appeared to follow these structural findings.

Her first depressive episode occurred immediately after the injury. In 2018, she experienced what was later understood as a probable hypomanic episode marked by compulsive shopping focused on items of particular colors, substantial work impairment that led to a transfer, and only fragmentary recall afterward. She also reported marked postpartum irritability after the birth of her daughter, who was four years old at the time of the current presentation. Interpersonal stressors had repeatedly worsened her mood, and ongoing relationship conflict coincided with the onset of her most recent depressive relapse in mid-2025. Her maternal grandmother had a history of bipolar-spectrum symptoms. The diagnosis was made clinically from longitudinal history, collateral family information, and treatment course. No structured clinical interview for DSM Disorders, Mini-International Neuropsychiatric Interview, Young Mania Rating Scale, or formal cognitive test was administered, so the diagnostic formulation remains probable rather than definitive.

She first presented in the current episode on 21 October 2025 after two months of insomnia that had not improved with melatonin. She described constant catastrophizing, relentless rumination, and a persistent sense that something bad was about to happen. Sleep was particularly disturbed. On nights when she could not sleep, she often felt paradoxically energetic, with racing thoughts centered on unfinished tasks. Two days before the visit, she had experienced child-like voices commenting on what she was doing. The available chart notes placed these experiences around sleep onset, and they were therefore managed clinically as hypnagogic phenomena rather than established psychosis. However, the record did not document duration, level of insight, dissociative context, trauma triggers, behavioral response, or impact on safety in enough detail to fully exclude trauma-linked intrusions or psychotic symptoms. Her Patient Health Questionnaire-9 (PHQ-9) score was 22, indicating severe depressive symptoms, and her Generalized Anxiety Disorder-7 (GAD-7) score was 14, consistent with moderate anxiety [15,16]. At that time, her medication regimen included sodium valproate 300 mg nightly, risperidone 0.5 mg nightly, lemborexant 2.5 mg nightly, alprazolam or zolpidem as needed for sleep, and Deanxit, containing flupentixol 0.5 mg plus melitracen 15 mg, one tablet daily, usually taken at night.

At follow-up on 5 November 2025, her PHQ-9 score had already fallen to 15, although she reported feeling worse overall. This improvement in PHQ-9 score occurred before dextromethorphan and piracetam were started and therefore cannot be attributed to CGR. She had been more irritable that week due to a relationship conflict. She also continued to experience hypnagogic phenomena around sleep onset. At this visit, glutamatergic augmentation was initiated using CGR, defined here as low-dose dextromethorphan with piracetam alongside CYP2D6 inhibition [11-13]. Dextromethorphan 30 mg nightly and piracetam 600 mg nightly were added, and valproate was increased to 500 mg nightly. Deanxit was continued, providing presumed CYP2D6 inhibition; this was not confirmed by genotype, plasma dextromethorphan/dextrorphan levels, or a formal pharmacokinetic assessment. Despite the lower PHQ-9 score, her subjective distress remained high.

By early December, the picture had changed in a noticeable but mixed way. She described feeling more stable and less mentally stuck, yet she also developed episodes of inappropriate laughter that gave her affect a somewhat moria-like quality. The available record describes the laughter as inappropriate and unmotivated in context, but it does not contain enough phenomenological detail to determine whether it represented anxious laughter, frontal moria, hypomanic activation, or a combination of these. Clinically, it was treated as a mild activation or overshoot signal because it emerged after dextromethorphan 30 mg and resolved after dose reduction. She had reduced her dextromethorphan dose on her own to 22.5 mg nightly because the 30 mg dose made her feel too elevated. At the same visit, piracetam was increased to 1,200 mg nightly. After that adjustment, her mood became more even. The inappropriate laughter subsided, and she no longer had the self-giggling that had worried her. Her PHQ-9 score remained 15, but both her own account and the clinician’s impression suggested a real change in the quality of her daily life. She felt less trapped in rumination and better able to engage with ordinary activities. No dissociative symptoms or worsening psychotic phenomena were noted at that stage.

Over the following months, her course continued to improve, although not in a straight line. By late January 2026, her PHQ-9 score had fallen to 10 and her GAD-7 score to 11. She had started keeping a journal, something she had not done in years, and family members said that she seemed less emotionally labile. She also reported feeling less anxious at work and said that the constant background sense of impending doom had faded. Around that time, however, she began to notice occasional short-term memory lapses and mild dissociative experiences, especially vivid or unusual dreams. These symptoms were considered likely to be multifactorial, with possible contributions from the overall medication burden, the baseline effects of frontal injury, and the glutamatergic agents themselves. Piracetam was, therefore, reduced back to 600 mg nightly, and the cognitive complaints eased without loss of mood stability. In late January, low-dose quetiapine 12.5 mg nightly was also added to support sleep and mood. No bedside cognitive screen or formal neuropsychological assessment was performed, so the cognitive complaints cannot be objectively quantified.

By late February and into March, her overall condition remained better, with PHQ-9 scores ranging from 10 to 12 and GAD-7 scores from 10 to 13. Two new issues appeared during this period. First, she developed delayed menses, which was attributed clinically to risperidone, so risperidone was replaced with aripiprazole 2.5 mg nightly. Second, travel and continued interpersonal stress triggered brief periods of low mood and avoidance. In March, paroxetine-controlled release 6.25 mg nightly and pregabalin 50 mg nightly were added for residual anxiety and sleep problems. Benzodiazepine exposure had also become a concern, as alprazolam and zolpidem had been prescribed repeatedly since October, and the patient herself remarked that she was taking “too many Xanax.” The addition of controlled-release paroxetine created a second pharmacokinetic period because it is a potent CYP2D6 inhibitor. This likely changed dextromethorphan exposure, but the magnitude and direction of that change cannot be determined without plasma drug/metabolite levels.

By April 2026, six months after the initial presentation, the patient said that her sleep was clearly better and that her mood was more stable, with less day-to-day fluctuation. She had resumed regular exercise and had started motorcycle riding lessons, both of which represented meaningful functional recovery in the context of longstanding post-TBI emotional dysregulation. Her PHQ-9 score was 10, and her GAD-7 score was 8. Residual episodes of low mood still occurred, especially during interpersonal stress, travel, or the days before menses, but these were now managed with as-needed dextromethorphan 15 to 30 mg and piracetam 600 to 1,200 mg rather than with fixed daily dosing of the CGR. The exact number of PRN doses per week or month, and the quantitative symptom change within 24-72 hours after each PRN dose, were not recorded. There was no return of the severe depressive state or the hypomanic symptoms seen in late 2025. Across the six-month period, no auditory hallucinations or other psychotic symptoms recurred beyond the initial hypnagogic phenomena, which had resolved early. After piracetam was reduced, the remaining cognitive side effects were mild and manageable.

Taken as a whole, the course suggested that initiating CGR on top of a regimen that already included presumed CYP2D6 inhibition coincided with a clinically important shift in a patient whose illness showed clear post-TBI and mixed-state features. Later stability likely reflected the combined effects of the glutamatergic agents, subsequent medication changes, and lifestyle recovery rather than any single intervention alone. She remained in regular outpatient follow-up, with ongoing attention to dosing, adverse effects, and psychosocial triggers. Monitoring documented in the clinical course included attention to mood activation, sleep, perceptual symptoms, dissociation, cognition, sedative exposure, and medication overuse risk. No formal DXM-specific safety protocol was attached to the chart (Table 1).

**Table 1:** Longitudinal assessment and treatment summary across induction, stabilization, and PRN maintenance phases. ALP: alprazolam; ARI: aripiprazole; BD: twice daily; CBD: cannabidiol; CR: controlled-release; DXM: dextromethorphan; DNX: Deanxit (flupentixol 0.5 mg/melitracen 15 mg); LEM: lemborexant; PGB: pregabalin; PIR: piracetam; PRN: pro re nata; PRO: propranolol; QTP: quetiapine; RIS: risperidone; SE: side effects; SRX: paroxetine controlled-release; STM: short-term memory; TRZ: triazolam; VPA: sodium valproate; ZOL: zolpidem; S: severe; MS: moderately severe; M: moderate; Mi: mild; PHQ-9: Patient Health Questionnaire-9; GAD-7: Generalized Anxiety Disorder-7.

Visit date	PHQ-9 (/27)	GAD-7 (/21)	Clinical notes	Current medication regimen (daily dose)
Phase I: Induction (October-December 2025)				
21 October 2025	22 (S)	14 (M)	Severe depressive relapse after two months of insomnia unresponsive to melatonin. Constant catastrophizing, relentless rumination, persistent sense of impending doom. On sleepless nights: paradoxical energy with racing thoughts about unfinished tasks. Two days prior: child-like auditory hallucinations/commenting-type experiences documented around sleep onset and later treated clinically as hypnagogic phenomena. Commenced mood stabilizer and low-dose antipsychotic. The later PHQ-9 dropped from 22 to 15, preceded by CGR initiation.	VPA 300 mg, RIS 0.5 mg, LEM 2.5 mg, TRZ 0.25 mg, ALP 0.25 mg/ZOL PRN, DNX 1 tab
05 November 2025	15 (MS)	12 (M)	PHQ-9 improved but patient reported feeling subjectively worse. Increased irritability after discovering partner’s suspected infidelity. Continued hypnagogic phenomena. DXM 30 mg and PIR 600 mg commenced as glutamatergic augmentation. VPA increased to 500 mg. DNX continued, with presumed but unmeasured CYP2D6 inhibition. LEM discontinued.	VPA 500 mg, RIS 0.5 mg, DXM 30 mg, PIR 600 mg, TRZ 0.25 mg, ALP 0.25 mg/ZOL PRN, DNX 1 tab
02 December 2025	15 (MS)	13 (M)	Feeling more stable, less mentally stuck, better engagement with daily activities. Transient mild hypomanic overshoot: inappropriate laughter with moria-like quality. Patient self-reduced DXM from 30 mg to 22.5 mg ("felt too elevated"). PIR increased to 1200 mg. After adjustment: mood more even, inappropriate laughter resolved. No dissociative symptoms or worsening psychotic phenomena. PRO added for tremor/anxiety. FHx confirmed (maternal grandmother, bipolar-spectrum).	VPA 500 mg, RIS 0.5 mg, DXM 22.5 mg, PIR 1200 mg, PRO 10–20 mg PRN, TRZ 0.25 mg, ALP 0.25 mg/ZOL PRN, DNX 1 tab
Phase II: Stabilization (December 2025-February 2026)				
30 December 2025	15 (MS)	12 (M)	Commenced therapeutic journaling. Less rumination, more positive cognition, reduced emotional rigidity. Improvement corroborated by family members. Resolution of hypomanic features. Vivid/unusual dreams. Began experiencing STM lapses and mild dissociative experiences (multifactorial: medication burden, frontal injury, glutamatergic agents). PIR adjusted to BD dosing.	VPA 500 mg, RIS 0.5 mg, DXM 22.5 mg, PIR 600 mg BD, PRO 10–20 mg PRN, TRZ 0.25 mg, ALP 0.25 mg/ZOL PRN, DNX 1 tab
27 January 2026	10 (M)	11 (M)	Substantial improvement. Anxiety significantly reduced, particularly at work. Background sense of impending doom faded. Persistent STM difficulties; patient self-adjusted PIR to half tablet nocte. PIR reduced to 600 mg; cognitive complaints eased without loss of mood stability. QTP 12.5 mg added for sleep/mood augmentation. No formal cognitive testing was performed.	VPA 500 mg, RIS 0.5 mg, QTP 12.5 mg, DXM 22.5 mg, PIR 600 mg, PRO 10–20 mg PRN, TRZ 0.25 mg, ALP 0.25 mg/ZOL PRN, DNX 1 tab
25 February 2026	12 (M)	13 (M)	Mood slightly low. Avoidance behaviour upon return to family home (Taiwan). Continued relationship tension. Delayed menses attributed to RIS; premenstrual mood deterioration. Excessive benzodiazepine use noted (patient remarked on over-reliance). PRO discontinued.	VPA 500 mg, RIS 0.5 mg, QTP 12.5 mg, DXM 22.5 mg, PIR 600 mg, TRZ 0.25 mg, ALP 0.25 mg/ZOL PRN, DNX 1 tab
Phase III: Consolidation and PRN maintenance (March-April 2026)				
25 March 2026	11 (M)	10 (M)	Persistent insomnia, using CBD adjunctively. Premenstrual mood deterioration. Patient stated “too many Xanax.” Major regimen change: RIS discontinued due to amenorrhoea and replaced by ARI. SRX added, creating a later potent CYP2D6-inhibition period. PGB added for residual anxiety/insomnia. ALP and ZOL discontinued. DXM/PIR restructured as low-dose regular plus PRN for stress-related and premenstrual dips.	VPA 500 mg, SRX 6.25 mg, ARI 2.5 mg, QTP 12.5 mg, PGB 50 mg, DXM 15 mg (+30 mg PRN), PIR 600 mg (+1200 mg PRN), TRZ 0.25 mg, DNX 1 tab
22 April 2026	10 (M)	8 (Mi)	Sleep clearly improved. Mood more stable with less day-to-day fluctuation. Resumed regular exercise and commenced motorcycle riding lessons. Residual low mood during relationship stress, travel, or premenstrual periods managed with PRN DXM/PIR. No recurrence of auditory hallucinations or psychotic symptoms since initial presentation. No hypomanic features since Dec 2025. Residual cognitive side effects mild and manageable. Regimen continued unchanged. Exact PRN frequency and 24–72-hour quantitative response were not recorded.	VPA 500 mg, SRX 6.25 mg, ARI 2.5 mg, QTP 12.5 mg, PGB 50 mg, DXM 15 mg (+30 mg PRN), PIR 600 mg (+600 mg PRN), TRZ 0.25 mg, DNX 1 tab

## Discussion

One point needs to be clarified at the outset. The drop in PHQ-9 score from 22 to 15 had already occurred by the 5 November 2025 visit, which was the same visit at which dextromethorphan and piracetam were started. That means the early numerical improvement cannot be credited to the glutamatergic regimen. The more relevant temporal signals came later: her own report of reduced rumination and greater mental flexibility within weeks, the appearance of mild hypomanic or disinhibition-like features at the 30 mg dextromethorphan dose, and the restoration of stability when the dose was lowered to 22.5 mg. These observations support only a temporal association, not proof that dextromethorphan or piracetam caused the improvement.

The baseline presentation also deserves careful attention. Before any glutamatergic medication was added, the patient had not only low mood and insomnia but also bursts of energy on sleepless nights, racing thoughts about unfinished tasks, marked irritability, and occasional child-like voices documented around sleep onset. In a patient with documented right frontal atrophy, that pattern is more consistent with a mixed or agitated depressive state within a probable post-TBI bipolar-spectrum formulation than with straightforward unipolar depression. Frontal injury is known to weaken inhibitory control and leave limbic circuits more easily activated [3,4]. In that setting, even modest NMDA blockade might plausibly reduce the “stuck” quality of depression while also pushing a partly disinhibited system toward mild activation. However, this remains a clinical interpretation rather than a demonstrated mechanism.

The dosing sequence offers further clues, but those clues should be interpreted cautiously. Benefit and overshoot appeared together at a dose of 30 mg of dextromethorphan. When the patient lowered the dose herself to 22.5 mg, and piracetam was increased, stability returned without loss of the earlier subjective gains. This pattern suggests that dextromethorphan was the medication most closely temporally linked to both the antidepressant-like effect and the transient activation. Piracetam, in contrast, may have acted as a modulator, helping to support downstream plasticity or allowing efficacy at a lower dextromethorphan exposure. This interpretation is hypothesis-generating only. It is not possible to separate the effects of dextromethorphan, piracetam, valproate titration, risperidone, Deanxit, sleep agents, psychosocial change, or later aripiprazole, paroxetine, pregabalin, and quetiapine from the available data.

Across six months, the course unfolded in three fairly distinct clinical phases in this patient. The first was an induction phase marked by daily dosing, rapid subjective benefit, and a brief hypomanic or disinhibition-like overshoot. The second was a stabilization phase in which dose adjustment restored euthymia. The third was a consolidation and maintenance phase, during which other agents were added, including aripiprazole, paroxetine-controlled release, pregabalin, and low-dose quetiapine, and lifestyle recovery also progressed through resumed exercise and other routines. By April 2026, she was using dextromethorphan and piracetam mainly as needed during predictable dips related to relationship stress, travel, or the premenstrual period. This should be described as an observed pattern in one patient, not as a validated three-phase model. Exact PRN frequency, adherence, and symptom response within 24-72 hours were not recorded, limiting reproducibility.

Pharmacokinetic limitations are central to interpretation. Early DXM exposure occurred in the setting of Deanxit and presumed CYP2D6 inhibition, whereas later exposure occurred after the introduction of paroxetine-controlled release, a potent CYP2D6 inhibitor [17]. These two periods likely differed in the dextromethorphan/dextrorphan ratio and total exposure. Because no CYP2D6 genotyping or plasma dextromethorphan/dextrorphan measurement was performed, the actual exposure associated with benefit, activation, dissociation, or cognitive complaints remains unknowable. Therefore, the apparent dose-response relationship should be interpreted as a clinical observation rather than as a pharmacokinetic conclusion.

Safety and misuse risk also require emphasis. The patient herself expressed concern about excessive alprazolam exposure, and benzodiazepine/sedative use was reduced later in the course. Although PRN dextromethorphan/piracetam was clinically useful in this case, PRN use in patients with TBI requires careful monitoring for escalating use, compulsive redosing, dissociation, perceptual changes, sleep disruption, and mood activation. This is especially important when frontal injury may contribute to impulsivity or impaired self-monitoring.

Hypothesis-generating molecular context. A broader biological context comes from recent cross-disorder transcriptomic work, but this evidence is indirect and not patient-specific. Using 2025 Genome-Wide Association Study (GWAS) summary statistics for major depressive disorder, bipolar disorder, and obsessive-compulsive disorder, meta-analyzed across six GTEx v8 brain regions, Cheung [14] reported different molecular signatures across these conditions. Major depressive disorder showed stronger negative enrichment for mitochondrial and oxidative phosphorylation pathways, whereas bipolar disorder showed disruption of telomere-maintenance pathways and signals related to plasticity and energetic stress. Because this TWAS was not performed in the present patient and remains separate from the clinical observation, it cannot establish the mechanism of response. At most, it provides a hypothesis that post-TBI bipolar-spectrum illness may involve a labile plasticity system that could be sensitive to glutamatergic modulation.

Several limitations affect interpretation. This was a single observational case drawn from a retrospective chart review, so causal claims must remain modest. Polypharmacy increased after the first two months, making later attribution difficult, especially because paroxetine-controlled release was added and is a potent CYP2D6 inhibitor [17]. The exact balance between standing and PRN dosing after March 2026 cannot be confirmed without the original handwritten records. No plasma dextromethorphan-to-dextrorphan ratios were measured, and no CYP2D6 genotyping was done, so the pharmacokinetic assumptions remain untested. No serum valproate levels were available, limiting interpretation of mood-stabilizer adequacy. No Young Mania Rating Scale (YMRS) [18] or standardized mania scale was administered, limiting characterization of the mild hypomanic or disinhibition-like overshoot. No formal cognitive screening or neuropsychological testing was performed, limiting interpretation of the reported memory and dissociative symptoms. The original neuroimaging could not be digitally reproduced or formally summarized from an accessible radiology report. Functional recovery also occurred alongside exercise, routine, and some psychosocial stabilization, so the effect of the glutamatergic agents cannot be isolated from these changes.

Even with those limits, the case has practical implications. It suggests that low-dose nocturnal dextromethorphan plus piracetam, added on top of possible CYP2D6 inhibition and mood-stabilizing cover, may be associated with a clinically meaningful change in a post-TBI bipolar-spectrum patient who had not improved enough with standard treatment. Early monitoring for hypomanic or disinhibition-like overshoot is important, as is readiness to lower the dose if activation emerges. Mood-stabilizer support at the outset also appears prudent. Once the patient has stabilized, shifting to carefully monitored PRN use during predictable triggers may reduce overall exposure while preserving a rescue option for state-sensitive relapses. These observations are hypothesis-generating and require a prospective, controlled study before any broader recommendation can be justified.

**Figure 1:**
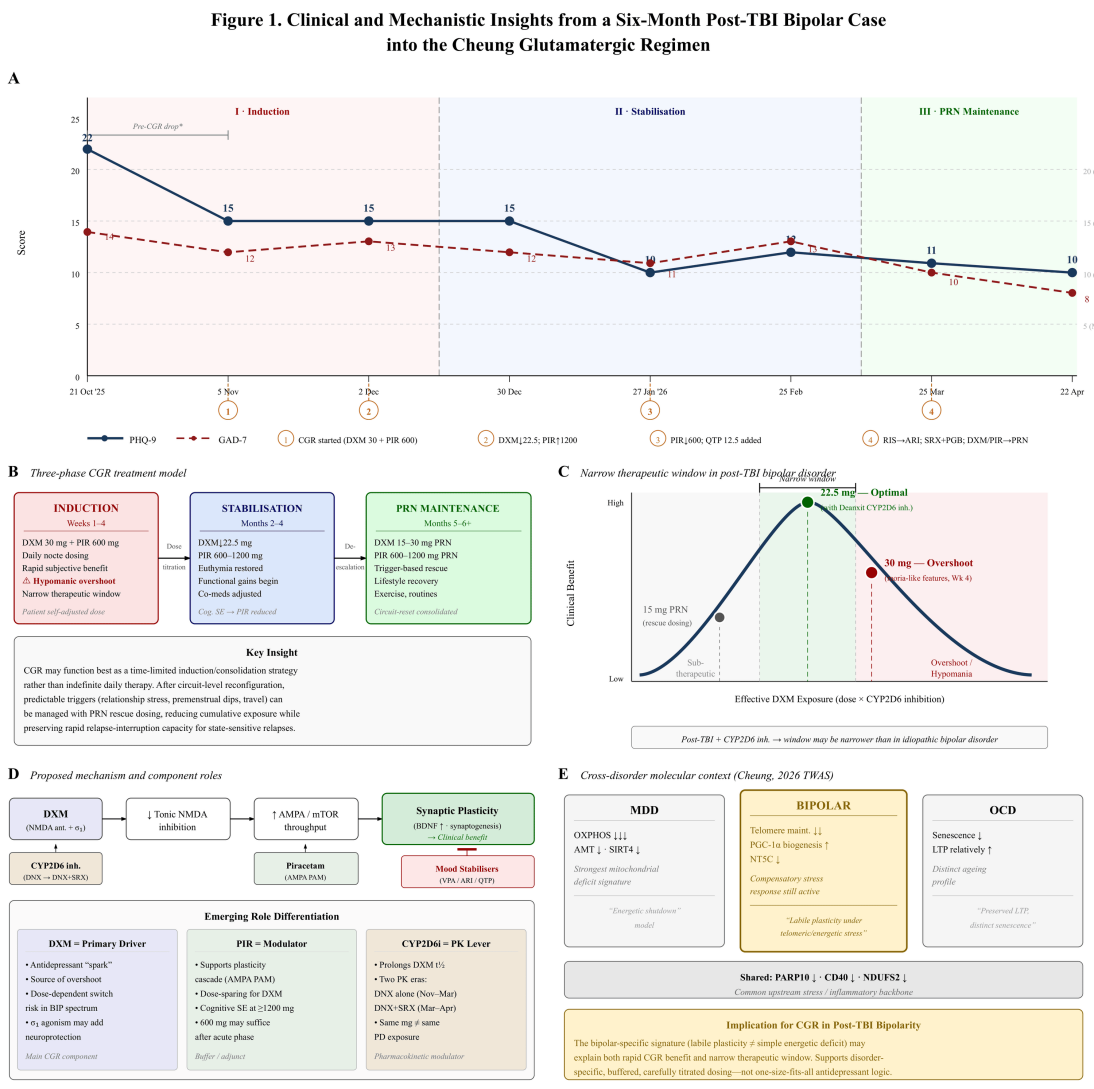
Key clinical and mechanistic insights from a six-month post-TBI bipolar case into the Cheung Glutamatergic Regimen (CGR). (A) PHQ-9/GAD-7 trajectories, October 2025-April 2026. Shading shows phases; numbered markers show medication milestones. The PHQ-9 fall from 22 to 15, preceded by CGR, and is not attributable to DXM/PIR; PHQ-9 ≥10 has 88% sensitivity/specificity for major depression [15]. (B) Observed single-patient pattern, not a validated model: induction with narrow therapeutic window and brief hypomanic/disinhibition-like overshoot; titration-led stabilization; PRN maintenance during predictable triggers. (C) Schematic DXM dose-response under CYP2D6 inhibition: benefit appeared at 22.5 mg and overshoot at 30 mg; pharmacokinetics were not confirmed. (D) Hypothesized roles: DXM as primary NMDA/σ1 signal, PIR as AMPA modulator, CYP2D6 inhibitors as PK levers, and mood stabilizers as an overshoot brake. Paroxetine CR in March 2026 marks a second PK era. These are hypotheses, not causal findings. (E) Indirect TWAS context: bipolar disorder may show telomere-maintenance/plasticity signals distinct from MDD OXPHOS suppression and OCD LTP/senescence patterns [14]; these findings are not patient-specific. ARI: aripiprazole; BIP: bipolar disorder; BDNF: brain-derived neurotrophic factor; CGR: Cheung Glutamatergic Regimen; DXM: dextromethorphan; DNX: Deanxit; LTP: long-term potentiation; MDD: major depressive disorder; OCD: obsessive-compulsive disorder; OXPHOS: oxidative phosphorylation; PAM: positive allosteric modulator; PGB: pregabalin; PIR: piracetam; PK: pharmacokinetic; PRN: pro re nata; QTP: quetiapine; SE: side effects; SRX: paroxetine controlled-release; TBI: traumatic brain injury; TWAS: transcriptome-wide association study; VPA: sodium valproate; PHQ-9: Patient Health Questionnaire-9; GAD-7: Generalized Anxiety Disorder-7. Credit: Artwork created using Microsoft PowerPoint.

## Conclusions

This six-month observational course shows that low-dose oral dextromethorphan combined with piracetam, added to ongoing CYP2D6 inhibition and mood-stabilizing treatment, was associated with meaningful and sustained clinical improvement in a patient with probable post-TBI bipolar-spectrum illness who had only partially responded to conventional therapy. Three points stand out. First, the early drop in PHQ-9 score occurred before the glutamatergic regimen was started and should not be attributed to it. Second, the baseline presentation was more consistent with a mixed or agitated depressive state in the context of frontal TBI than with pure bipolar depression, which may help explain both the quick subjective improvement and the narrow therapeutic window. Third, dextromethorphan appeared temporally linked to both benefit and activation, while piracetam may have functioned as a modulator that supported stabilization at a lower dose and later de-escalation to PRN use.

These conclusions remain limited by the single-case design, retrospective chart review, absence of structured diagnostic and mania ratings, lack of serum valproate levels, lack of formal cognitive testing, inaccessible imaging reports, unmeasured CYP2D6 status and DXM/dextrorphan levels, later polypharmacy, and incomplete PRN-frequency data. When considered alongside recent cross-disorder transcriptomic findings, the case supports only the hypothesis that these dynamics may reflect bipolar-spectrum plasticity abnormalities rather than a generic antidepressant mechanism. Low-dose oral glutamatergic augmentation may therefore represent a practical, mechanistically informed option for this difficult clinical population, provided that dosing is individualized, early monitoring is careful, misuse/escalation risk is addressed, and the regimen is used as an induction and consolidation tool rather than assumed to be lifelong daily therapy. Prospective studies with standardized diagnostic assessment, YMRS, cognitive screens, serum valproate levels, CYP2D6 genotyping, plasma DXM/dextrorphan measurement, and quantified PRN response windows are needed.
